# Assessing central serous chorioretinopathy with deep learning and multiple optical coherence tomography images

**DOI:** 10.1038/s41598-022-05051-y

**Published:** 2022-02-03

**Authors:** Junseo Ko, Jinyoung Han, Jeewoo Yoon, Ji In Park, Joon Seo Hwang, Jeong Mo Han, Kyu Hyung Park, Daniel Duck-Jin Hwang

**Affiliations:** 1 Department of Applied Artificial Intelligence, Sungkyunkwan University, Seoul, Korea; 2 RAON DATA, Seoul, Korea; 3 Lux Mind, Incheon, Korea; 4 Department of Medicine, Kangwon National University Hospital, Kangwon National University School of Medicine, Chuncheon, Gangwon-do South Korea; 5 Seoul Plus Eye Clinic, Seoul, Korea; 6 Kong Eye Center, Seoul, Korea; 7 Department of Ophthalmology, Seoul National University Bundang Hospital, Seongnam, Korea; 8 Department of Ophthalmology, Hangil Eye Hospital, 35 Bupyeong-daero, Bupyeong-gu, Incheon, 21388 Korea; 9 Department of Ophthalmology, Catholic Kwandong University College of Medicine, Incheon, Korea

**Keywords:** Machine learning, Medical imaging

## Abstract

Central serous chorioretinopathy (CSC) is one of the most common macular diseases that can reduce the quality of life of patients. This study aimed to build a deep learning-based classification model using multiple spectral domain optical coherence tomography (SD-OCT) images together to diagnose CSC. Our proposed system contains two modules: single-image prediction (SIP) and a final decision (FD) classifier. A total of 7425 SD-OCT images from 297 participants (109 acute CSC, 106 chronic CSC, 82 normal) were included. In the fivefold cross validation test, our model showed an average accuracy of 94.2%. Compared to other end-to-end models, for example, a 3D convolutional neural network (CNN) model and a CNN-long short-term memory (CNN-LSTM) model, the proposed system showed more than 10% higher accuracy. In the experiments comparing the proposed model and ophthalmologists, our model showed higher accuracy than experts in distinguishing between acute, chronic, and normal cases. Our results show that an automated deep learning-based model could play a supplementary role alongside ophthalmologists in the diagnosis and management of CSC. In particular, the proposed model seems clinically applicable because it can classify CSCs using multiple OCT images simultaneously.

## Introduction

Central serous chorioretinopathy (CSC) is the fourth most common retinopathy and is characterized by the serous detachment of the neurosensory retina^1^. If subretinal fluid (SRF) persists, it can damage the outer layer of the retina, causing permanent loss of visual function, which is associated with a decrease in the patient’s quality of life^2,3^. CSCs are usually classified into acute or chronic CSC according to the chronicity of the disease, and it is important to evaluate the chronicity of the disease to determine the treatment plan or prognosis^4^.

CSC has traditionally been diagnosed using multimodal imaging modalities, including fluorescein angiography (FA) and indocyanine green angiography (ICGA)^4,5^. Among these modalities, optical coherence tomography (OCT) is non-invasive, fast, and shows highly reproducible results^6,7^ and is now considered a gold-standard imaging modality for the follow-up of CSC patients^5^.

Applying deep learning to OCT images has been demonstrated to be useful for assessing CSC. The prior work reported that the proposed deep learning model can distinguish between 1) normal and CSC and 2) acute and chronic CSC types for a given OCT image^8^. While the prior work provided valuable insight into the potential of using an OCT image to assess CSC, it may not have considered actual clinical practice where multiple OCT images, instead of one image, from a single patient are analyzed at the same time. In clinical practice, ophthalmologists usually make a final diagnosis by simultaneously looking at several OCT images per case. Therefore, in this study, we aimed to build a deep learning model that could comprehensively read multiple OCT images. In addition, the performance of our proposed model for discriminating between normal vs. acute CSC vs. chronic CSC was investigated and compared with that of ophthalmologists.

## Results

A total of 7425 images from 297 participants (normal 82, CSC 215) were included in the study. The 215 patients with CSC were enrolled at the outpatient clinic, with 109 and 106 being diagnosed with acute and chronic CSC, respectively. The mean age of the participants in the normal group was 64.3 ± 8.25 years and that of those in the CSC group was 54.12 ± 9.88 years. Detailed information on the data used in this study is presented in Table 1.

**Table 1:** Baseline characteristics of patients who had undergone macular OCT.

	Normal	CSC		
		Acute CSC	Chronic CSC	Total CSC
Image, no	2050	2725	2650	5375
Patients, no	82	109	106	215
Age (years), mean (SD)	64.30 (8.25)	49.31 (8.36)	59.7 (8.83)	54.12 (9.88)
**Sex, no (%)**				
Male	22 (26.83%)	95 (87.16%)	97 (91.50%)	192 (89.30%)
Female	60 (73.17%)	14 (12.84%)	9 (8.50%)	23 (10.79%)
**Eye, no. (%)**				
Right	42 (51.22%)	62 (56.88%)	22 (20.75%)	84 (39.07%)
Left	40 (48.78%)	47 (43.12%)	84 (79.25%)	131 (60.93%)

*OCT* optical coherence tomography, *CSC* central serous chorioretinopathy, *SD* standard deviation.

### Model performance

As shown in Table 2, the proposed model for classifying multiple classes (i.e., acute, chronic, and normal), combined with ResNet-50 and logistic regression, showed an average cross-validation accuracy of 94.2% (95% CI 0.897–0.986), sensitivity of 94.9%, and specificity of 99.1%. The kappa score between our model and the ground truth was 0.909. The proposed model showed the best accuracy compared to other possible combinations such as VGG19 + logistic regression (92%), VGG19 + SVM (90%), and VGG19 + XGBoost (89%). In addition, our proposed model had a higher accuracy than both the three-dimensional convolutional neural network (3D-CNN) (75.6%) and CNN-long short-term memory (CNN-LSTM) (83%) models.

**Table 2:** Accuracy of cross-validation in baseline models and the proposed model.

	Onefold	Twofold	Threefold	Fourfold	Fivefold	Average
3D-CNN	80%	**82%**	79%	73%	64%	75.6%
CNN-LSTM	80%	79%	**86%**	85%	78%	83%
VGG19 + XGB	**92%**	89%	91%	87%	86%	89%
VGG19 + SVM	92%	**93%**	90%	95%	80%	90%
VGG19 + Logistic Regression	94%	**95%**	90%	93%	88%	92%
ResNet-50 + Logistic Regression	91%	95%	97%	**98%**	90%	**94.2%**

Significant values are in bold.

*3D-CNN* three-dimensional convolutional neural network, *CNN-LSTM* convolutional neural network–long short-term memory model, *VGG19* VGG architecture with 19 layers and our custom fully connected layers, *ResNet-50* ResNet architecture with 50 layers, *XGB* XGBoost Classifier, *SVM* support vector machine.

### Model performance in comparison with ophthalmologists

**Figure 1:**
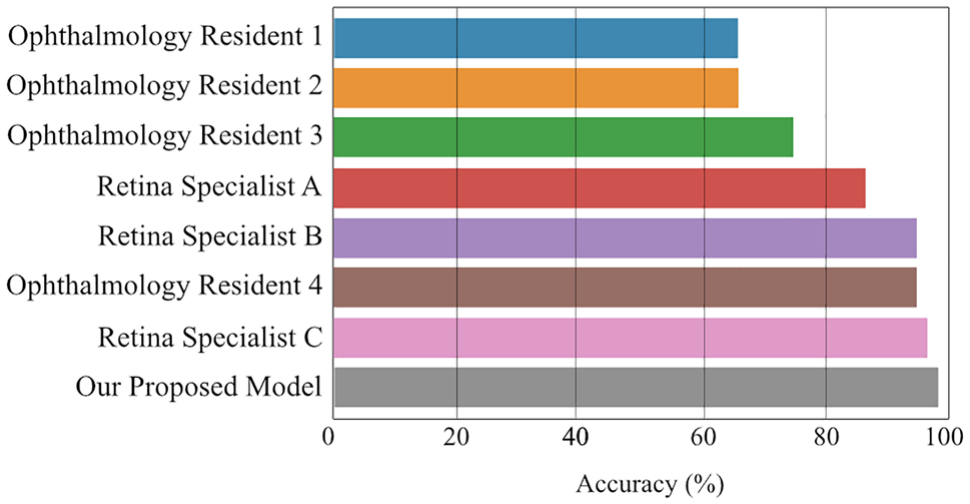
Performance comparison between ophthalmologists and the proposed model. This figure shows the accuracy between the ophthalmologists and the proposed model in distinguishing different CSC subtypes. Our proposed model showed the highest accuracy compared to any ophthalmologist. Our model achieved a high accuracy of 98.33%, whereas the ophthalmologists showed accuracies between 66 and 96.66%.

**Figure 2:**
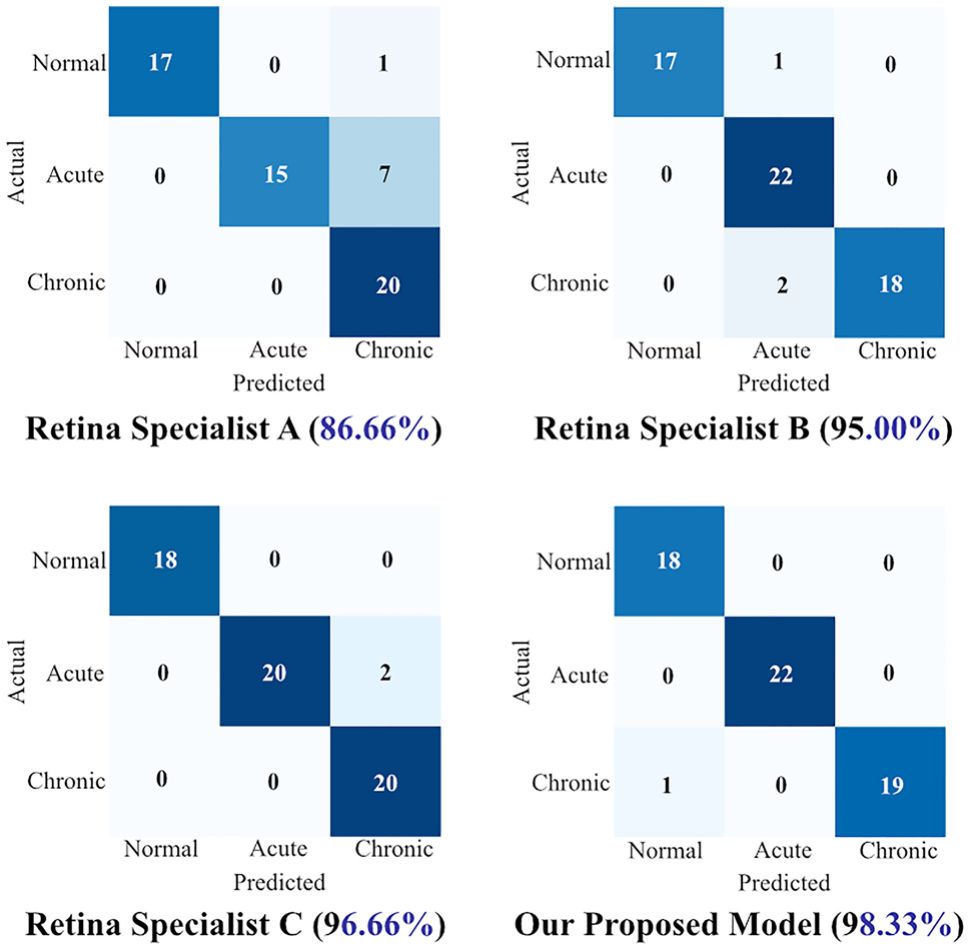
Confusion matrix comparison between our proposed model and the three retina specialists. The confusion matrix shows the confusion matrix of the 4th fold from the fivefold cross-validation. The x-axis denotes the predicted class and the y-axis denotes the actual class of a given patient. Our proposed model showed 98.3% accuracy and accurately classified all cases except for one normal case. All the retina experts had more than 10 years of clinical experience.

To evaluate the proposed model from a clinical perspective, we selected the 4th fold which showed the best performance result among the fivefold cross-validation, and provided the data to the seven ophthalmologists. Figure 1 shows the performances of the seven ophthalmologists and our proposed model. Our model achieved a higher accuracy than any ophthalmologist, implying that the proposed model exhibited expert-level performance. Our proposed model achieved a high accuracy of 98.33%, whereas the ophthalmologists showed accuracies between 66.00 and 96.66%. Figure 2 shows the confusion matrix of the 4th fold of the proposed model and retina specialists. The proposed model performed better than all the ophthalmologists and accurately classified all cases except one. In nine cases, incorrect classifications were noted for three or more ophthalmology residents out of four. Among these nine cases, all three ophthalmology experts correctly diagnosed three cases. Our model also correctly classified these three cases that were correctly classified by the ophthalmology experts. A difficult case that was wrongly classified by three ophthalmology residents and two of the three experts was correctly classified by the model. Table 3 illustrates the examples of consensus and non-consensus cases between the model and graders.

**Table 3:** Examples of consensus and non-consensus cases between residents, specialists, and our models.

									
Ground truth	Acute	Acute	Acute	Acute	Acute	Acute	Acute	Acute	Normal
Resident 1	Chronic	Chronic	Chronic	Chronic	Chronic	Chronic	Chronic	Chronic	Normal
Resident 2	Chronic	Chronic	Chronic	Chronic	Chronic	Chronic	Chronic	Chronic	Chronic
Resident 3	Chronic	Chronic	Chronic	Chronic	Chronic	Chronic	Chronic	Chronic	Chronic
Resident 4	Chronic	Acute	Acute	Acute	Acute	Acute	Acute	Acute	Chronic
Specialist A	Chronic	Acute	Chronic	Chronic	Chronic	Acute	Chronic	Chronic	Normal
Specialist B	Acute	Acute	Acute	Acute	Acute	Acute	Acute	Acute	Normal
Specialist C	Acute	Acute	Acute	Chronic	Acute	Acute	Acute	Acute	Normal
Proposed model	Acute	Acute	Acute	Acute	Acute	Acute	Acute	Acute	Normal

*Acute* Acute central serous chorioretinopathy, *Chronic* Chronic central serous chorioretinopathy.

## Discussion

CSC usually shows spontaneous improvement in more than half of patients, but it can lead to the permanent deterioration of visual function due to damage to the photoreceptor cells of the outer retinal layer^4,9^. Therefore, it is important to distinguish acute CSC from chronic CSC considering clinical information such as the time of symptom onset. Acute CSC usually has a self-limited natural course. However, if there is no spontaneous improvement after follow-up, active treatment such as focal laser treatment, intravitreal anti-vascular endothelial growth factor injection, or photodynamic therapy may be considered. On the other hand, in the case of chronic CSC, it may be difficult to improve visual function in cases with permanent photoreceptor damage. If an active lesion such as subretinal fluid is observed, active intervention should be performed immediately to prevent further damage to visual function.

In this study, we built a model to assess CSC using multiple OCT images simultaneously. Unlike the prior work^8^ that only used one image per patient, the proposed model considered a real clinical scenario in which several OCT images per case were used in diagnosis. Our model effectively distinguished between acute and chronic CSC, and its performance was comparable to or better than those of 3D-CNN, CNN-LSTM, and experienced ophthalmologists.

In our experiments, the proposed model outperformed the ophthalmologists in assessing CSC. There were nine cases where three of four ophthalmology residents failed to correctly classify the images, and all three ophthalmology experts correctly diagnosed three cases among these 9 cases. Our model correctly classified all nine cases, demonstrating that the proposed model can perform at the expert level. Furthermore, there was a case in which three residents and two experts incorrectly classified the condition. However, our model could correctly classify such a challenging case. Thus, the proposed model showed promising performance for classifying CSCs using deep learning and 25 spectral domain (SD)-OCT images. We expect our model to play a supportive role alongside ophthalmologists. In addition, our model should help to diagnose challenging cases for non-retinal specialists.

We evaluated the proposed model using 3D-CNN^10^ and CNN-LSTM^10^ models. These models comprise two parts: (i) extracting a feature description using multiple images and (ii) making a final decision using the extracted feature vector. In the CNN-LSTM model, multiple two-dimensional (2D) images were used as input, and a description of each image was generated in the CNN layers. After the CNN layers, each LSTM cell classifies diseases using the sequential information of images. Similar to the CNN-LSTM model, the proposed model used a CNN model to extract a description vector for each image. To further improve the performance, our model used lesion cuts and a softmax output. In the 3D-CNN model, a 3D image consisting of a set of multiple 2D images was used as an input. The fully connected layer in the 3D-CNN model could classify diseases using the description vector generated from the 3D-CNN layers.

While 3D-CNN and CNN-LSTM models require much training data, only a relatively small number of SD-OCT images were available in this study. To remedy this issue, we split the model decision process into two parts: (i) the prediction of a single image and (ii) the final decision. First, we trained the single-image prediction (SIP) model using only the lesion cuts. Thereafter, the final decision (FD) classifier was trained using logistic regression, and it could learn with a relatively small amount of training data. Thus, the proposed model showed good performance in assessing CSCs without large training data.

This study has several limitations. First, in our study, the number of subjects was not large enough and external validation was not performed. External validation should be performed with images from different OCT manufacturers in future studies because all the images were extracted from a single OCT device at one academic center. However, the dataset was sufficient to demonstrate the feasibility of our model for classifying CSCs with multiple OCT images simultaneously. Second, we investigated the performance of the model without clinical information (e.g., symptom duration, age, sex) or other imaging modalities such as fundus photography, infrared reflectance imaging, FA, and ICGA. Performance obtained by combining diverse clinical data with multiple modalities would be more accurate compared to considering only OCT images. Third, the proposed model may suffer from training complexity. In other words, lesion cuts were annotated by ophthalmologists to train the SIP model, which could introduce additional cost to the process. In addition, the output of the SIP model was used to train the FD classifier. This means that training the SIP and FD classifiers cannot be parallelized. In future work, we plan to enhance our model to achieve an end-to-end model, which can train the feature descriptor and final classifier together. Regardless of the above limitations, the proposed model shows promising performance, and further investigations seem necessary regarding its potential impact on clinical practice.

In summary, we developed a deep learning model that showed good performance in distinguishing chronic CSC from acute CSC simultaneously using multiple SD-OCT images. Thus, our model could play a supplementary role alongside ophthalmologists in the classification of CSC. Additionally, automation of the classification process using the model may improve patients’ quality of life by improving prognosis and may save cost and time for both healthy people and patients with CSC.

## Methods

This study was conducted in accordance with the 1964 Helsinki Declaration. The Ethics Committee of Hangil Eye Hospital approved the research protocols and their implementation. The committee waived the requirement for obtaining informed consent, given that this was a retrospective observational study of medical records and was retrospectively registered.

### Data collection and labelling

We analyzed the records of patients who visited Hangil Eye Hospital between January 2017 and January 2021. We used SD-OCT (Heidelberg Spectralis, Heidelberg Engineering, Heidelberg, Germany) images of normal participants and patients with CSC. Central volume scans using a 25-scan pattern and macular thickness map protocols were routinely performed using the SD-OCT scanner at our hospital. Through this process, a volumetric assessment of the central retinal structures consisting of 25 single horizontal axial scans was routinely performed (scanning area: 6 × 6 mm, centered at the fovea). Although horizontal or vertical scan images were also available, they were not used in this study. Instead, we only used the central volume scans comprising 25 images.

All CSC cases were diagnosed by fundus examination, FA, ICGA, and OCT images by independent retinal specialists (DDH and JSH). A confocal scanning laser ophthalmoscope (Heidelberg Retina Angiograph, HRA; Heidelberg Engineering, Germany) was used to perform simultaneous FA and ICGA in all CSC cases. One eye per patient was selected for this study, with one visit per patient. Based on the retinal pigment epithelium or photoreceptor status and the duration of symptoms, patients were classified as having acute CSC or chronic CSC as follows: (1) acute CSC with SRF lasting ≤ 4 months and (2) chronic atrophic CSC with definite retinal pigment epithelium and photoreceptor atrophy with or without SRF. In cases of disagreement, a third retina specialist (JMH) evaluated the discrepancy and discussed the case with other specialists. After discussion, all discrepancies were resolved by consensus. Our analysis excluded data that showed the presence of other potentially conflicting retinal pathologies such as age-related macular degeneration, polypoidal choroidal vasculopathy, pachychoroid neovasculopathy, and pachychoroid pigment epitheliopathy.

### Data preprocessing

To feed the SD-OCT images into deep neural networks, we first cropped the 596 × 1264-pixel SD-OCT images and obtained 380 × 764-pixel red–green–blue (RGB) images. We then down-sampled the cropped image to 163 × 254-pixel RGB images. To remedy overfitting issues, we also performed a data augmentation process in which the model learns from randomly transformed input images, including horizontal image flips, random brightness changes from 0.7 to 1.3, and random rotations of the image of up to 15°. The data augmentation process was applied only during the training phase.

### The proposed model

**Figure 3:**
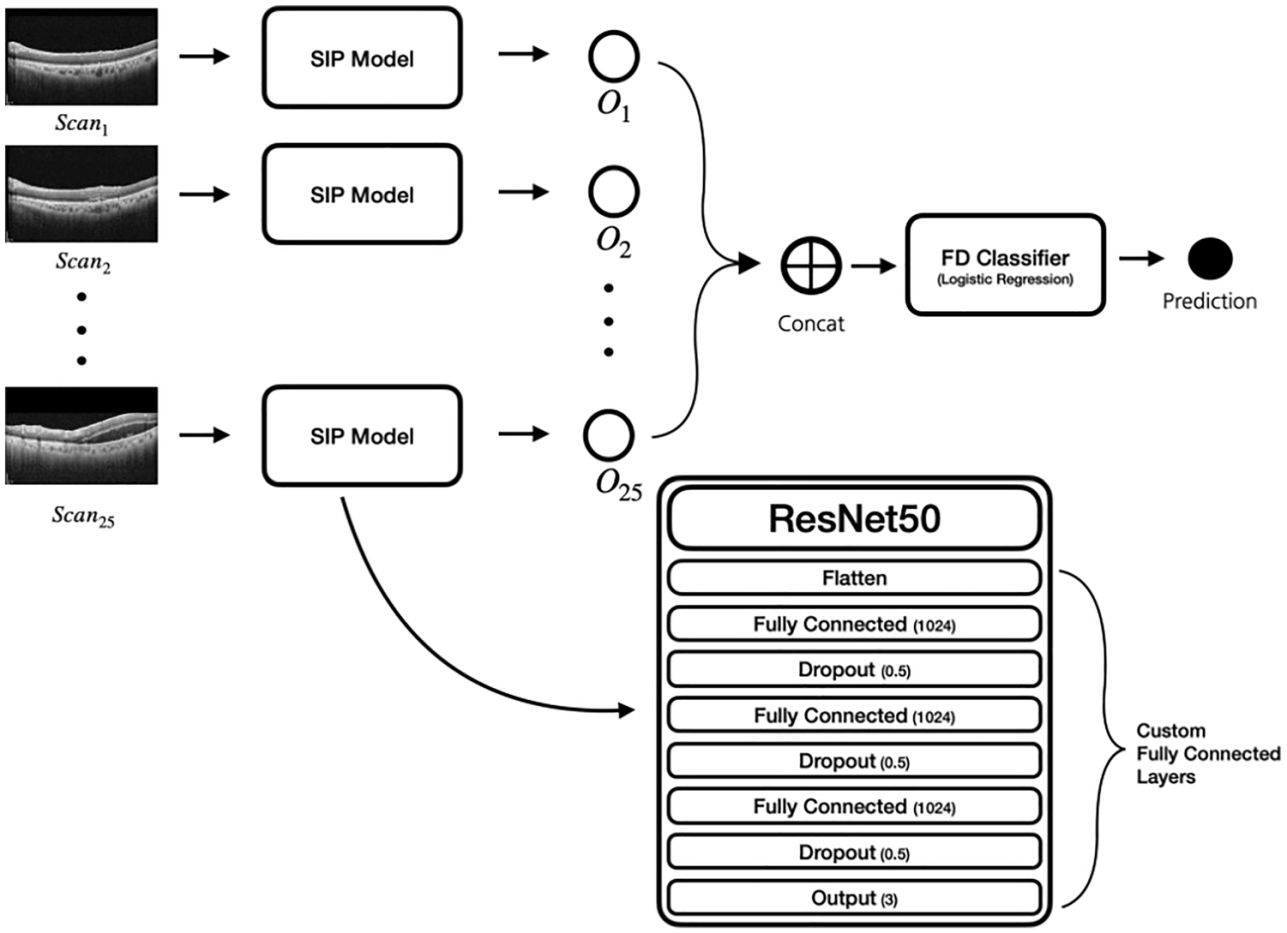
An illustration of our proposed model. We used ResNet-50 as a single-image prediction (SIP) model and stacked custom fully connected layer after the convolution layers. For the final decision (FD) classifier, logistic regression was used.

To classify each subject with a set of 25 SD-OCT images into three different classes (i.e., acute CSC, chronic CSC, and normal), we built a hierarchical deep learning model that consists of two different modules: SIP and FD classifier. As shown in Fig. 3, for the SIP model, we utilized a popular CNN architecture, ResNet-50^11^, to extract the softmax score (i.e., class probability) from a single SD-OCT image. We used other well-known CNN-based models including VGG-16^12^ and Inception V3^13^, but we decided to use ResNet-50^11^ as it performed better than the others. More specifically, we stacked four linear layers with dropouts on top of the ResNet-50 architecture. We used the final linear layer with a softmax activation function to predict the multiclass classification result.

To train the SIP model, we first initialized ResNet-50 with pre-trained weights that were acquired from the large-scale image dataset ImageNet^11,14^. We then froze the ResNet-50 layers and only updated the weights of the four fully connected layers during training. This deep learning technique, called transfer learning^15^, helped our model to avoid overfitting and train faster^15^. In the test phase, we use normal 25 SD-OCT scans as the input. In the training phase of the SIP model, however, we extracted only lesion cuts from all 25 SD-OCT images. The purpose of the SIP model is to classify a single OCT image into each class. If non-lesion cuts are used in the training phase, the SIP model cannot distinguish between normal-class images and non-lesion cuts from CSC images. To train the SIP model for its purpose, we only used lesion cuts in the training phase to achieve improved model performance. The output of the SIP model is a three-dimensional softmax vector (e.g., [0.2, 0.2, 0.6]), where each scalar value denotes the class probability for acute CSC, chronic CSC, and normal, respectively. By concatenating all softmax vectors for the 25 SD-OCT images, we passed a 25 × 3 (i.e., number of SD-OCT cuts for the patient × number of classes)-shaped matrix to the FD classifier.

For the FD classifier, we used logistic regression^16,17^ which took a 25 × 3 shaped matrix extracted from the previous stage as input and predicted the final class label (i.e., acute CSC, chronic CSC, or normal). We tried other well-known machine learning classifiers such as XGBoost^18^, SVM^19^, and Random Forest^20^, but we opted for logistic regression as it performed better than others. The FD classifier made overall clinical decisions based on 25 softmax vectors extracted from a single patient.

### Experiment setup

#### Baseline models: 3D-CNN and CNN-LSTM

**Figure 4:**
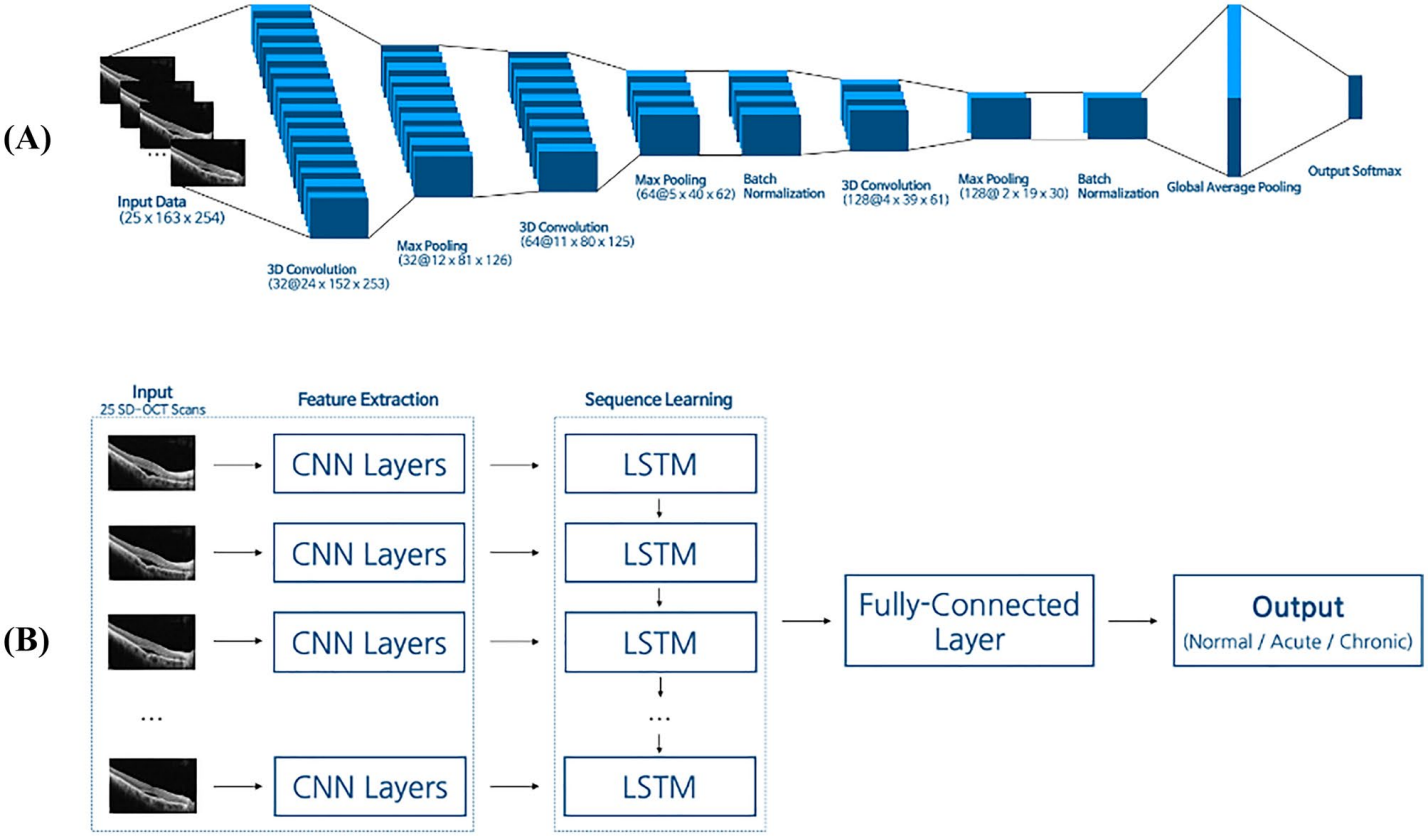
An illustration of the baseline models (CNN-LSTM, 3D-CNN). (**a**) The 3D Convolution module extracted spatial and temporal features from the 25 SD-OCT cuts. The last fully connected layer classified each class using a 3D image description vector extracted from the 3D convolution module. The number in front of ‘@’ denotes the filter size, and after the ‘@’ is the size of the output vector. (**b**) The CNN-LSTM (Convolutional Neural Network—Long Short Term Memory) model used CNN as the feature extractor, LSTM for sequence learning, and the fully connected layer as a classifier. The CNN architecture summarized a single image into a latent vector. LSTM analyzed each latent vector from each image. The final classifier predicted the labels using outputs of LSTM. The LSTM layers contain 64 cells. Acute and chronic in the output box indicate acute CSC and chronic CSC, respectively.

To compare the performance of our proposed model with those of other deep neural networks, we tested the 3D-CNN^21^ and CNN-LSTM^10^ models (Fig. 4). All baseline models took the same input images as the proposed model; each image was a 163 × 254-pixel RGB image. The 3D-CNN model used as a baseline model in this study took a 4-dimensional image set (i.e., number of images × image width × image height × number of channels) as inputs and predicted the final class label (i.e. acute CSC, chronic CSC, and normal). As shown in Fig. 4a, the overall architecture consisted of three 3D convolution layers, three max pooling layers, two batch normalization layers, a global average pooling layer, and a fully connected layer followed by a softmax activation function. The 3D-CNN model considered the series (volume) of the 25 SD-OCT images simultaneously and performed the convolution.

Figure 4b shows the overall architecture of the CNN-LSTM model used in this study. The embedding vectors extracted from 25 2D convolution blocks were fed into LSTM layers with 64 hidden units, which were followed by a fully connected layer with a softmax activation function. Each 2D convolution block consisted of six 2D convolutional layers with max-pooling layers. By combining CNN and LSTM, the model could consider both spatial and sequential information^22^.

#### The proposed model

To train and evaluate the proposed model, fivefold cross-validation was performed. We first split the entire dataset into five different folds, and then trained the model with four folds and tested it with the remaining fold. Note that each fold has similar distribution to the original data distribution. The detailed description of the fivefold cross-validation information is summarized in Table 4. The same patient was not included in the training and test sets at the same time. The SIP model was trained using 75% of all cases in the training set, which was randomly sampled similar to the distribution of the training data. The FD classifier was trained using the remaining 25% cases that were not used in the SIP model. For the FD classifier, we used the softmax values, which were the prediction results of the 25 SD-OCT cuts by the SIP model.

**Table 4:** Description of the fivefold cross-validation split.

	1 Fold	2 Fold	3 Fold	4 Fold	5 Fold
Total cases	64	56	58	60	59
Normal	18	16	15	18	15
Acute CSC	22	21	22	22	22
Chronic CSC	24	19	21	20	22

*CSC* central serous chorioretinopathy.

To evaluate the proposed model from a clinical perspective, we selected the classification round that showed the best performance result among the fivefold cross-validation and provided the test fold data of the chosen round to seven ophthalmologists as follows: three retina specialists with more than 10 years of clinical experience each at an academic ophthalmology center, and four ophthalmology residents.

## Data Availability

The data are not available for public access because of patient privacy concerns, but are available from the corresponding author upon reasonable request.
